# Free Triiodothyronine Serves as a Potential Predictor of Long-Term Heart Failure Following Acute Myocardial Infarction: A Single-Center Follow-Up Study in China

**DOI:** 10.1155/crp/6649022

**Published:** 2025-11-01

**Authors:** Xinying Ye, Meihong Shi, Senyang Chen, Jiarui Shen, Zhiqian Chen, Lukun Guo, Kaizheng Gong, Pei Zhao

**Affiliations:** ^1^Department of Cardiology, Institute of Cardiovascular Disease, Yangzhou Key Lab of Innovation Frontiers in Cardiovascular Disease, Affiliated Hospital of Yangzhou University, Yangzhou University, Yangzhou, Jiangsu, China; ^2^Home Ward, Taizhou Hospital of Zhejiang Province Affiliated to Wenzhou Medical University, Linhai, Zhejiang, China

**Keywords:** free triiodothyronine, heart failure, myocardial infarction, predictive value

## Abstract

**Background:**

This study explored the potential role of FT3 in predicting long-term heart failure (HF) in patients with acute myocardial infarction (AMI), so as to provide relevant information about the Chinese population.

**Methods:**

This was an observational, retrospective, single-center study of consecutive patients with AMI enrolled at the Affiliated Hospital of Yangzhou University. The patients were divided into the HF group or the non-HF group according to the occurrence of HF after AMI. Cox proportional hazards regression models identified factors independently associated with long-term HF. The patients were segregated into two groups by the median level of FT3 (4.63 pmol/L): the Group 1 (< 4.63 pmol/L) and the Group 2 (> 4.63 pmol/L), and the Kaplan–Meier survival analysis was used to estimate the HF-free survival between the two groups. The receiver operating characteristic (ROC) curves were used to evaluate the predictive performance of FT3 on long-term HF among patients with AMI.

**Results:**

A total of 269 AMI patients were included. Multivariable Cox regression analysis indicated that age (*p* < 0.001), FT3 (*p*=0.030), and LVEF (*p* < 0.001) were independent prognostic factors for long-term HF after AMI. The Kaplan–Meier survival analysis revealed a significantly lower HF-free survival rate in patients with lower FT3 levels (*p* < 0.01). The ROC analysis revealed that FT3 exhibited good predictive performance for long-term HF after AMI, with an AUC of 0.736 (*p* < 0.01).

**Conclusions:**

Lower levels of FT3, even within the normal range, not only serve as independent risk factors for long-term HF after AMI but also predict a higher incidence of it.

## 1. Introduction

Heart failure (HF) is a critical endpoint at the terminal stage of various heart diseases with high rates of mortality and rehospitalization, and acute myocardial infarction (AMI) is a major cause of HF [1, 2]. Epidemiological investigations suggest that modern medicine can recognize AMI early and perform early reperfusion therapy. Despite a reduction in short-term mortality associated with AMI, the long-term hazards associated with HF and mortality are still increasing [3–5]. Early identification of individuals at high risk is crucial for improving their quality of life by preventing or delaying the onset of long-term HF. Additionally, it is crucial for the success of therapeutic interventions.

Thyroid hormones possess cardioprotective effects, including anti-inflammatory, anti-apoptotic, and antifibrotic capacities. Even slight changes in serum thyroid hormone concentrations can impact cardiovascular function [6]. Reduced levels of free triiodothyronine (FT3) have been strongly correlated with increased mortality rates among critically ill patients, indicating the severity of their conditions. Moreover, it is important to note that FT3 levels can fluctuate during acute episodes of illness [7]. Previous studies have suggested a correlation between low FT3 levels and a poor prognosis in HF patients [8, 9]. However, the relationship between FT3 levels and the development of HF after AMI remains unclear. Therefore, this study offers insights into the Chinese cohort by examining the relationship between FT3 levels and the long-term risk of HF in patients with AMI at a single center in China.

## 2. Materials and Methods

### 2.1. Study Population

**Figure 1:**
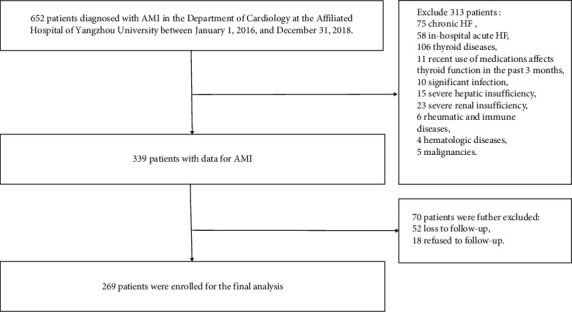
Flow diagram of study participants.

This retrospective study involved a total of 269 patients who were admitted to the Department of Cardiology at the Affiliated Hospital of Yangzhou University between January 1, 2016, and December 31, 2018, and were diagnosed with AMI. The study cohort consisted of 207 male patients and 62 female patients, with an average age of 64.2 years. The diagnosis of AMI was made by cardiologists following clinical guidelines [10]. The diagnostic criteria included the presence of typical clinical symptoms of chest pain, dynamic changes observed in the electrocardiogram, and elevated levels of myocardial injury markers, with at least two of three conditions being met. Exclusion criteria were applied to exclude patients with chronic HF or in-hospital acute HF, thyroid diseases, recent use of medications that could affect thyroid function within the past 3 months, significant infection, abnormal liver or renal function, rheumatic and immune diseases, hematologic diseases, malignancies, loss to follow-up, or refusal to participate in the follow-up. The inclusion of the study population is shown in Figure 1.

### 2.2. Data Collection

Clinical data of the study subjects were obtained from the hospital's electronic medical records system, including past medical history, laboratory test results, echocardiographic findings, interventional procedures, and prescribed medications. In this study, the normal range of FT3 was defined as 3.28–6.47 pmol/L. The peak CK-MB and peak troponin I values were determined as the maximum values among multiple measurements.

### 2.3. Definitions and Outcomes

**Figure 2:**
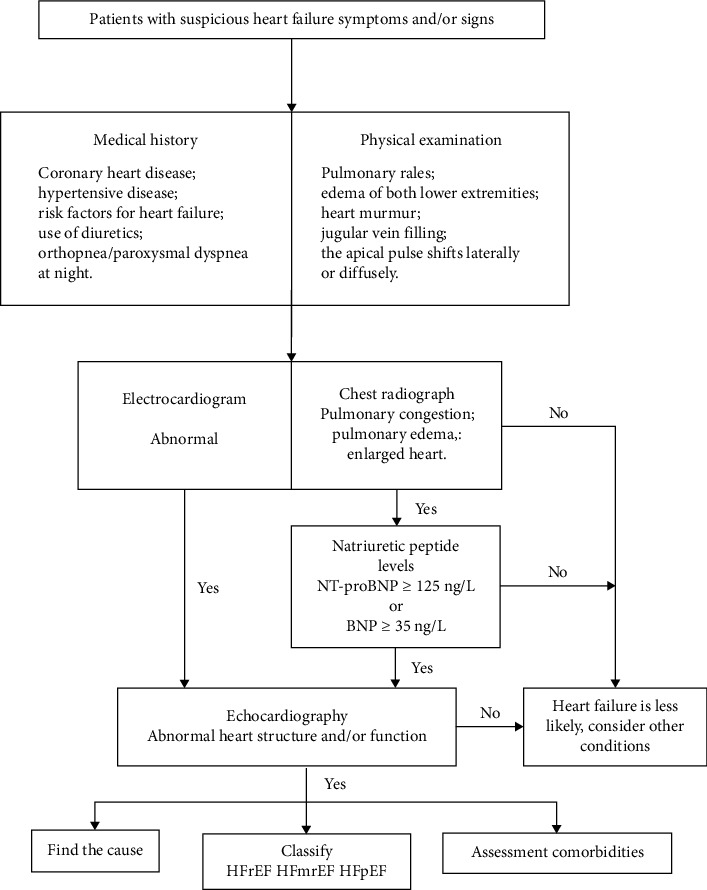
Heart failure diagnostic algorithm. Abbreviations: NT-proBNP: N-terminal B-type natriuretic peptide, BNP: B-type natriuretic peptide, HFrEF: heart failure with reduced ejection fraction, HFmrEF: heart failure with midrange ejection fraction, HFpEF: heart failure with preserved ejection fraction.

In this study, the diagnostic algorithm for HF in patients with AMI was referred to the 2018 Chinese Guidelines [11] (Figure 2). Acute HF can be effectively ruled out if the levels of B-type natriuretic peptide (BNP) are below 100 ng/L or N-terminal BNP (NT-proBNP) are below 300 ng/L. Similarly, chronic HF can be ruled out if BNP is below 35 ng/L or NT-proBNP is below 125 ng/L. In the diagnosis of acute HF, the categorization of NT-proBNP levels should consider patient's age and renal function: NT-proBNP levels exceeding 450 ng/L for patients under 50, 900 ng/L for patients over 50, 1800 ng/L for patients over 75, and above 1200 ng/L for those with renal insufficiency (glomerular filtration rate, eGFR < 60 μmol/L).

The primary endpoint was the first occurrence of HF. Follow-up data were collected from medical records, in-person interviews during clinic visits, or telephone interviews with patients and their families. Events were verified by physicians based on the guidelines [11]. The median follow-up duration was 39 months.

This study was conducted following the principles outlined in the Declaration of Helsinki (as revised in 2013). This study protocol was approved by the Research Ethics Committee of the Affiliated Hospital of Yangzhou University (approval number: 2022-YKL 06-28-004). Due to the noninterventional nature of this study, patients were exempted from signing an informed consent form.

### 2.4. Statistical Analysis

All analyses were conducted using SPSS 21.0 and R 4.1.3 (with the following R packages: tableone, survival, pROC, stringr). Continuous normally distributed variables were presented as means ± standard deviations and compared using Student's *t*-tests. Non-normally distributed variables were presented as median (interquartile range) and compared using the Mann–Whitney *U*-test. Categorical data were expressed as numbers (percentage) and compared using the chi-square test or Fisher's exact test. Univariable and multivariable Cox proportional hazards analyses were performed to identify variables associated with HF following AMI. The Kaplan–Meier curves were plotted to compare the cumulative incidence rates of HF between the HF group and the non-HF group, and the log-rank test was used for statistical comparison. The receiver operating characteristic (ROC) curves were constructed, and the area under the ROC curve (AUC) was calculated to evaluate the performance of FT3 levels in predicting long-term HF among patients with AMI.

All tests were two-sided, and *p* values < 0.05 were considered statistically significant.

## 3. Results

### 3.1. Baseline Characteristics

At the end of follow-up, these study subjects were divided into two groups based on the occurrence of HF after AMI: the HF group and the non-HF group. The patients in the HF group were significantly older compared to those in the non-HF group (60.4 years vs. 71.9 years; *p* < 0.05). However, no significant differences were observed between the two groups concerning gender, past medical history, heart rate, systolic blood pressure, or type of myocardial infarction (Table 1).

In our study, the established normal range for FT3 levels was 3.28–6.47 pmol/L. Of the participants, six individuals showed FT3 levels below this normal range. Compared to the non-HF group, patients in the HF group exhibited higher levels of creatinine (71 vs. 78.5 μmol/L; *p*=0.002) and D-dimer (0.2 vs. 0.3 mg/L; *p* < 0.001). Conversely, they displayed lower levels of hemoglobin (145.6 × 10^12^/L vs. 135.9 × 10^12^/L; *p* < 0.001), FT3 (4.7 vs. 4.2 pmol/L; *p* < 0.05), and left ventricular ejection fraction (LVEF) (65% vs. 62%; *p* < 0.05). Notably, despite the observed differences in LVEF, it remained within the normal range for both groups. There were no other significant differences between the two groups (Table 2).

A lower percentage of patients in the HF group underwent percutaneous coronary intervention (PCI) compared to those in the non-HF group (72.7% vs. 84%; *p* < 0.05). No significant difference in medication use was observed between the two groups (Table 3).

### 3.2. Factors Affecting Long-Term HF After AMI

Baseline variables with statistical significance (Tables 1, 2, and 3), as well as gender, type of myocardial infarction, total cholesterol, triglyceride, high-density lipoprotein cholesterol, low-density lipoprotein cholesterol, glycosylated hemoglobin, thyrotropin, and free thyroxine, were included in the univariate Cox regression analysis. This analysis identified that age, gender, hemoglobin, creatinine, D-dimer, FT3, LVEF, and PCI were associated with long-term HF after AMI.

The multivariate Cox regression analysis revealed that age (HR 1.072, 95% CI 1.044–1.101; *p* < 0.001), FT3 (HR 0.673, 95% CI 0.472–0.962; *p*=0.030), and LVEF (HR 0.953, 95% CI 0.931–0.975; *p* < 0.001) were independent predictors of long-term HF in patients with AMI (Table 4).

### 3.3. Effect of FT3 on Long-Term HF After AMI

**Figure 3:**
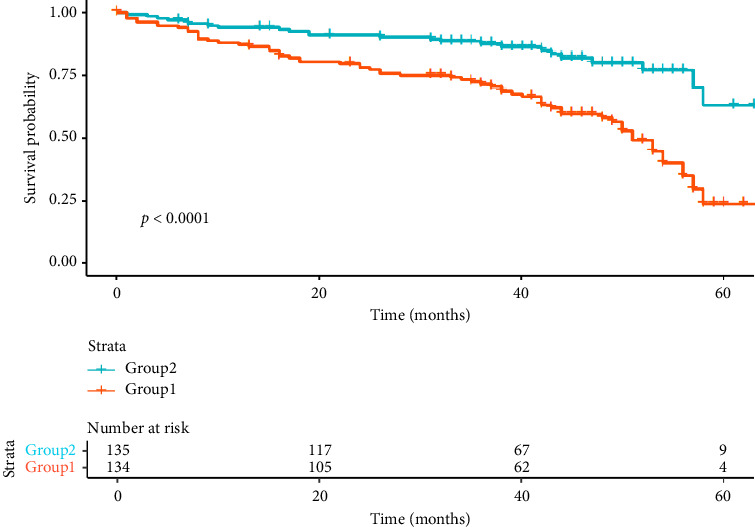
The Kaplan–Meier curves for HF-free survival rate in patients with AMI.

Based on the median FT3 level of 4.63 pmol/L, the patients were divided into Group 1 (FT3 < 4.63 pmol/L, *n* = 135) and Group 2 (FT3 > 4.63 pmol/L, *n* = 134). The incidence of long-term HF at the end of the follow-up period was 47.4% in the Group 1 and 17.9% in the Group 2, indicating a 2.64-fold higher risk of HF in the Group 1 compared to the Group 2. The Kaplan–Meier curves for HF-free survival in patients with AMI are shown in Figure 3. Significant differences in HF-free survival between the two groups were observed during the long-term follow-up: The HF-free survival rate was remarkably lower in the Group 1 compared to the Group 2 (22.5% vs. 62.9%, log-rank *p* < 0.001).

### 3.4. Predictive Value of FT3 for Long-Term HF After AMI

**Figure 4:**
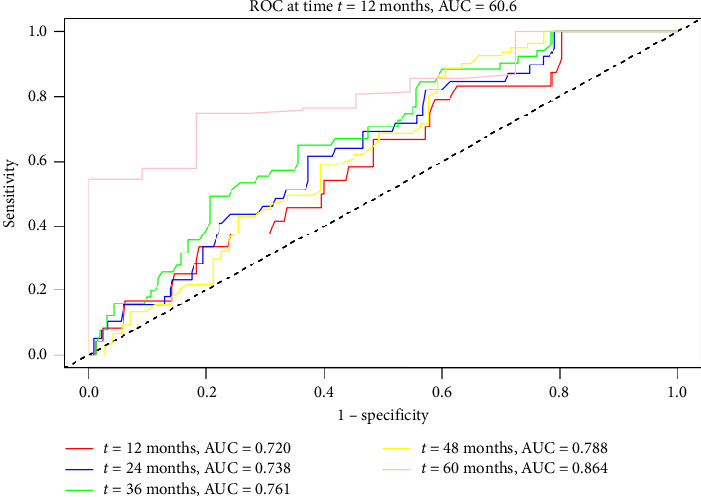
Time-dependent ROC curves of FT3 for predicting long-term HF after AMI.

The predictive value of FT3 for long-term HF after AMI is demonstrated in Figure 4. The ROC curve analysis showed that the sensitivity and specificity of FT3 in predicting long-term HF were 68.2% and 66.3% (AUC = 0.736, 95% CI 0.676–0.797; optimal cut-off value = 4.55 pmol/L). The time-dependent ROC curve analysis also revealed that FT3 had a certain predictive power for long-term HF following AMI. The AUC values at 12, 24, 36, 48, and 60 months were 0.720, 0.738, 0.761, 0.788, and 0.864, respectively (Figure 4).

## 4. Discussion

Low levels of FT3 are frequently linked to a poor prognosis in patients with cardiovascular disease. Nevertheless, the clinical importance of FT3 in individuals with heart disease, particularly in the setting of long-term HF following AMI, remains unclear due to a paucity of data specific to Chinese patients. Accordingly, the primary objective of this study was to investigate the relationship between FT3 levels and the occurrence of long-term HF among Chinese patients with AMI. The findings indicate that even lower FT3 levels within the normal range independently increase the risk of long-term HF after AMI. Moreover, serum FT3 levels may serve as a valuable prognostic indicator of long-term HF following AMI. These results underscore the potential importance of assessing FT3 levels as a simple yet informative approach to identifying individuals at risk for long-term HF in the context of AMI [12, 13]. Significantly, these insights contribute to enhancing the management and care of patients with AMI.

Post–myocardial infarction HF is classified into two subtypes: early postinfarction HF (present at admission for AMI or occurring during hospitalization) and long-term postinfarction HF (developing after discharge). The CREATE study in China reported an incidence of HF within 7 days among STEMI patients of 19.3% [14]. However, there is limited research on the occurrence of long-term HF after AMI. Studies conducted in Europe and America demonstrated that the incidence of HF between 30 days and 6 years following AMI ranged from 13.1% to 37.5% [15, 16]. In this study, we observed an HF incidence of 32.7% after AMI during a median follow-up of 39 months, which aligns with the findings in European and American populations.

The thyroid gland produces two primary hormones: thyroxine (T4) and triiodothyronine (T3). T4, the inactive form, is converted into the active form, T3, through the action of type-1 deiodinase and type-2 deiodinase. A small amount of T4 is also converted into inactive rT3 by type-3 deiodinase [6]. In cases of inflammation, hypoxia, and hemodynamic disturbance, the expression of type-3 deiodinase is upregulated, resulting in a decreased conversion of T4 into T3. It is widely accepted that the decrease in FT3 levels may be an adaptive response of the body to reduce catabolic metabolism and save energy expenditure [17]. However, numerous studies have reported a correlation between decreased FT3 levels and a poor prognosis. The patients with AMI may present with low FT3 levels, and severe cases may even experience low T3 syndrome (LT3S) [18]. A meta-analysis demonstrated that low T3 syndrome is an independent risk factor for poor cardiovascular prognosis, associated with an increased risk of all-cause death, cardiovascular death, and major adverse cardiovascular events (MACE) [19]. Su et al. found that AMI patients with low FT3 levels exhibited more severe myocardial injuries and higher inflammatory markers than those with normal FT3 levels [20]. Liao et al. [21] provided further evidence supporting a dose-dependent association between FT3 levels and mortality in patients with acute HF. Among the participants in this study, a total of 6 patients (2.2%) developed low T3 syndrome, and it was observed that all of them experienced HF, resulting in an incidence rate of 100%. The incidence rate in this subgroup was 3.1 times higher compared to the overall study cohort. Notably, the overall incidence of low T3 syndrome among AMI patients in this study was relatively low. This can be explained by the exclusion of critically ill patients and individuals with nosocomial HF, severe infection, liver disease, and kidney failure from the study population. Consequently, the overall disease severity of the study cohort was relatively mild.

Recent studies have investigated the association between FT3 levels and the prognosis of patients with AMI. Yamazaki et al. examined the impact of FT3 levels on the prognosis of long-term hemodialysis patients following AMI and found that lower FT3 levels were linked to a higher risk of MACE [22]. Additionally, Song et al. [13] and Wang et al. [23] reported that low FT3 levels are independent predictors of mortality and MACE in diverse patient populations, including those who underwent primary PCI and those who did not receive reperfusion therapy during the acute phase of AMI. In this study, our main objective was to examine the association between baseline FT3 levels and the occurrence of long-term HF in patients with AMI, including both those who received PCI therapy and those who did not. The results of our investigation revealed that low FT3 levels were an independent risk factor for long-term HF in patients with AMI, which is consistent with findings from previous studies. Alzavaios et al. [24] demonstrated that changes in T3 levels after AMI were correlated with early and late recovery of cardiac function, and that T3 levels at 6 months were an independent predictor of late functional recovery. Unfortunately, we were only able to measure FT3 levels at admission in our study. It is crucial to note that thyroid hormone levels decrease rapidly within 1 week after AMI [25]. Additionally, serum FT3 levels can indirectly reflect myocardial T3 levels [26]. While the majority of patients in our study cohort exhibited FT3 levels within the normal range, it is conceivable that individuals with lower FT3 levels may later develop low T3 syndrome.

The role of FT3 in the pathophysiology of AMI is multifaceted. FT3 affects heart rate, myocardial contractility, and other cardiac processes, exerting diverse effects on myocardial function through both nongenomic and genomic mechanisms [27, 28]. Studies conducted in experimental and clinical settings investigating low T3 syndrome have shown significant changes in cardiac structure and function [29]. Circulating levels of FT3 have also been associated with ventricular remodeling and reduced LVEF both in the early post-AMI period and during follow-up assessments. The effectiveness of exogenous FT3 supplementation in improving outcomes in AMI remains contentious [29]. Animal studies have demonstrated that administering exogenous T3 after AMI could provide myocardial protection [30], resembling the effects of beta-blockers [31], and it inhibits the conversion of T4 to T3. In a clinical trial conducted by Pingitore et al., the authors examined the effectiveness and safety of long-term oral synthetic T3 supplementation in 37 AMI patients diagnosed with low T3 syndrome. After a 6-month follow-up, the researchers observed that exogenous T3 supplementation not only increases FT3 levels in patients but also ameliorates dysfunction in the infarcted zone and enhances stroke volume [32]. Importantly, none of the patients experienced documented hyperthyroidism or arrhythmia during the study period. Other clinical trials suggest that T3 replacement therapy may partially increase cardiac output and reduce systemic vascular resistance [33–35], but its impact on outcomes remains inconclusive. In our study, despite the majority of AMI patients having FT3 level within the normal range, a lower FT3 level upon admission remained an independent risk factor for long-term HF subsequent to AMI. Further investigation is needed to determine whether AMI patients with normal-range FT3 level could benefit from exogenous T3 supplementation and its potential mechanisms. Importantly, the study found no significant difference in FT4 levels between patients with HF and those without HF following AMI. Because T4 is exclusively secreted by the thyroid gland and T3 is produced by both the thyroid gland and various other tissues, including the heart, the reduction in FT3 levels among AMI patients might indirectly indicate myocardial metabolic status, rather than serving as a direct therapeutic target.

## 5. Limitations

This study had several limitations. Firstly, it was a retrospective, observational study conducted at a single center. Secondly, this study was unable to assess FT3 levels during follow-up as they were not measured, leaving uncertainty about any potential changes in FT3 levels after AMI. Additional studies are required to investigate the relationship between dynamic changes in FT3 levels and long-term HF after AMI. Thirdly, serum FT3 levels may not precisely indicate myocardial thyroid hormone levels, which can decrease in the hearts of AMI patients due to hypoxia. Finally, this study did not consider the impact of atrial fibrillation and chronic obstructive pulmonary disease on long-term HF following AMI, and will be considered in future studies [36, 37].

## 6. Conclusion

In conclusion, our study found that a lower FT3 level, even within the normal range, independently contributes to long-term HF following AMI. Serum FT3 level could serve as a valuable predictor of long-term HF after AMI. These findings have important implications for the long-term medical management of patients with AMI.

## Figures and Tables

**Table 1:** Demographic and clinical characteristics of the two groups.

Characteristics	Total (*N* = 269)	Non-HF group (*N* = 181)	HF group (*N* = 88)	*t*/*χ*^2^/*Z*	*p* value
Age (years)	64.2 ± 12.4	60.4 ± 12.2	71.9 ± 8.8	−8.78	< 0.001
Male *n* (%)	207 (77.0%)	145 (80.1%)	62 (70.5%)	3.113	0.078^#^
Hypertension *n* (%)	188 (69.9%)	123 (68.0%)	65 (73.9%)	0.982	0.322^#^
Diabetes *n* (%)	83 (30.9%)	52 (28.7%)	31 (35.2%)	1.172	0.279^#^
Dyslipidemia *n* (%)	88 (32.7%)	58 (32%)	30 (34.1%)	0.113	0.737^#^
Previous MI *n* (%)	9 (3.3%)	5 (2.8%)	4 (4.5%)	—	0.48^#^
Smokers *n* (%)	123 (45.7%)	87 (48.1%)	36 (40.9%)	1.222	0.269^#^
HR (beats/min)	73.0 (65, 84)	73.0 (65.0, 80.5)	75 (66, 85)	−1.412	0.158
SBP (mmHg)	135 (120, 150)	136 (120, 150)	135 (120, 153.5)	−0.021	0.983
STEMI *n* (%)	166 (61.7%)	105 (58.0%)	61 (69.3%)	3.204	0.073^#^

*Note:* Values are expressed as mean ± SD or median (interquartile range) or number (%). STEMI: ST-segment elevation myocardial infarction.

Abbreviations: HR, heart rate; SBP, systolic blood pressure.

^#^For the chi-square test.

**Table 2:** Comparison of laboratory and equipment variables between the two groups.

Characteristics	Total (*N* = 269)	Non-HF group (*N* = 181)	HF group (*N* = 88)	*t*/*χ*^2^/*Z*	*p* value
WBC (10^9^/L)	8.6 (7.1, 10.6)	8.5 (7.1, 10.4)	8.7 (6.8, 10.6)	−0.342	0.733
Hemoglobin (10^12^ g/L)	142.4 ± 17.7	145.6 ± 16.7	135.9 ± 18.1	4.332	< 0.001
Platelet (10^9^/L)	188.7 ± 56.7	192.4 ± 52.2	181.0 ± 64.8	1.56	0.12
Hs-CRP (mg/L)	14.3 (5.1, 19.8)	13.8 (4.1, 20.4)	14.7 (6.2, 19.5)	−0.904	0.336
Albumin (g/L)	40.9 ± 4.2	41.3 ± 3.9	40.2 ± 4.8	1.954	0.052
ALT (U/L)	34.0 (25.5, 48.5)	34.0 (25.5, 48.5)	33.5 (25.2, 48.5)	−0.292	0.771
Blood glucose (U/L)	7.6 (6.2, 10.7)	7.3 (6.0, 0.4)	8.2 (6.4, 11.2)	−1.497	0.134
Serum creatinine (μmol/L)	72.0 (63.0, 86.0)	71.0 (62.0, 83.0)	78.5 (66.2, 93.2)	−3.033	0.002
Uric acid (μmol/L)	361.4 ± 94.9	361.4 ± 93.8	361.3 ± 97.8	0.009	0.993
Serum potassium (mmol/L)	3.9 (3.7, 4.2)	3.9 (3.7, 4.2)	3.9 (3.7, 4.2)	−0.531	0.596
CK-MB (U/L)	73 (31, 181)	70 (27, 163)	80 (35.7, 195.5)	−1.373	0.17
CTnI (U/L)	13 (4, 30)	10.0 (3.0, 30.0)	19.0 (5.0, 30.0)	−1.759	0.079
Cholesterol (mmol/L)	4.4 ± 1	4.5 ± 1.1	4.2 ± 0.8	1.894	0.059
Triglyceride (mmol/L)	1.7 (1.2, 2.4)	1.7 (1.2, 2.4)	1.7 (1.2, 2.5)	−0.425	0.671
HDL-C (mmol/L)	1 ± 0.2	1 ± 0.2	1 ± 0.2	−0.673	0.501
LDL-C (mmol/L)	2.4 ± 0.7	2.5 ± 0.8	2.4 ± 0.6	0.7	0.485
D-dimer (mg/L)	0.2 (0.1, 0.4)	0.2 (0.1, 0.3)	0.3 (0.2, 0.6)	−4.539	< 0.001
HbA1c (%)	7 (6.0, 8.2)	6.9 (5.9, 8.1)	7.0 (6.2, 8.4)	−1.606	0.108
TSH (mIU/L)	1.3 (0.7, 2.1)	1.2 (0.8, 2)	1.3 (0.6, 2.5)	−0.087	0.931
FT3 (pmol/L)	4.5 ± 0.6	4.7 ± 0.6	4.2 ± 0.5	6.848	< 0.001
FT4 (pmol/L)	11 (10, 12.)	11 (10, 12)	11 (9, 12)	−0.113	0.91
LAD	35 (32, 37)	35 (32, 37)	35 (33, 37.0)	−0.659	0.51
LVEDD	53 (50, 55)	53 (50.5, 55)	53 (50, 57.7)	−1.178	0.239
LVEF (%)	64 (60, 67)	65 (62, 69)	62 (56.2, 64)	−6	< 0.001

*Note:* Values are expressed as mean ± SD or median (interquartile range). ALT: glutamic-pyruvic transaminase; CK-MB: creatine kinase isoenzyme; CTnI: troponin I; HbA1C: glycosylated hemoglobin; Hs-CRP: hypersensitivity C-reactive protein; FT3: free triiodothyronine; FT4: free thyroxine.

Abbreviations: HDL-C, high-density lipoprotein cholesterol; LAD, left atrial diameter; LDL-C, low-density lipoprotein cholesterol; LVEDD, left ventricular end-diastolic diameter; LVEF, left ventricular ejection fraction; TSH, thyroid-stimulating hormone.

**Table 3:** Comparison of reperfusion treatment and medication between the two groups.

Characteristics	Total (*N* = 269)	Non-HF group (*N* = 181)	HF group (*N* = 88)	*t*/*χ*^2^/*Z*	*p* value
PCI *n* (%)	216 (80.3%)	152 (84.0%)	64 (72.7%)	4.737	0.03^#^
Medication at discharge *n* (%)					
Aspirin + clopidogrel	229 (85.1%)	153 (84.5%)	76 (86.4%)	0.157	0.692^#^
Aspirin + ticagrelor	45 (16.7%)	31 (17.1%)	14 (15.9%)	0.063	0.802^#^
ACEI/ARB	172 (63.9%)	116 (64.1%)	56 (63.6%)	0.005	0.942^#^
β-blockers	212 (78.8%)	139 (76.8%)	73 (83.0%)	1.345	0.246^#^
Statin drugs	266 (98.9%)	179 (98.9%)	88 (100%)	—	1^∗^
Regular medication *n* (%)	231 (85.9%)	157 (86.7%)	74 (84.1%)	3.343	0.558^#^

*Note:* Values are expressed as number (%) or median (interquartile range).

Abbreviation: PCI, percutaneous coronary intervention.

^∗^For Fisher's exact test.

^#^For the chi-square test.

**Table 4:** Univariate and multivariate Cox proportional hazards regression analysis of HF events.

Characteristics	Univariate		Multivariate	
	HR (95% CI)	*p*	HR (95% CI)	*p*
Age	1.086 (1.063, 1.110)	< 0.001	1.072 (1.044, 1.101)	< 0.001
Male	0.570 (0.360, 0.903)	0.017	0.735 (0.434, 1.254)	0.253
STEMI	1.379 (0.876, 2.170)	0.165	—	—
Hemoglobin	0.977 (0.966, 0.987)	< 0.001	1.001 (0.985, 1.016)	0.945
Cholesterol	0.849 (0.681, 1.058)	0.145	—	—
Triglyceride	0.950 (0.798, 1.132)	0.566	—	—
HDL-C	1.032 (0.533, 1.997)	0.926	—	—
LDL-C	0.993 (0.763, 1.293)	0.960	—	—
Serum potassium	1.012 (1.004, 1.020)	0.004	1.008 (0.999, 1.017)	0.097
D-dimer	1.148 (1.028, 1.281)	0.014	0.951 (0.832, 1.088)	0.466
HbA1c	1.036 (0.913, 1.175)	0.585	—	—
TSH	1.090 (0.980, 1.214)	0.113	—	—
FT3	0.441 (0.324, 0.599)	< 0.001	0.673 (0.472, 0.962)	0.030
FT4	0.938 (0.835, 1.053)	0.278	—	—
LVEF	0.938 (0.917, 0.959)	< 0.001	0.953 (0.931, 0.975)	< 0.001
PCI	0.615 (0.384, 0.985)	0.043	1.255 (0.733, 2.149)	0.408

*Note:* STEMI: ST-segment elevation myocardial infarction; FT3: free triiodothyronine; FT4: free thyroxine.

Abbreviations: CI, confidence interval; HDL-C, high-density lipoprotein cholesterol; HR, hazard ratio; LDL-C, low-density lipoprotein cholesterol; LVEDD, left ventricular end-diastolic diameter; LVEF, left ventricular ejection fraction; PCI, percutaneous coronary intervention; TSH, thyroid-stimulating hormone.

## Data Availability

The datasets used and analyzed during this study are available from the corresponding author upon reasonable request.
